# Clinical application of the AUC-guided dosage adjustment of docetaxel-based chemotherapy for patients with solid tumours: a single centre, prospective and randomised control study

**DOI:** 10.1186/s12967-020-02394-w

**Published:** 2020-06-08

**Authors:** Ning Sun, Bo Shen, Jiali Zhu, Xiaomei Zhang, Huayun Zhu, Geyu Liang, Deliang Yang, Jianwei Lu, Yan Zhang

**Affiliations:** 1 Department of Medical Oncology, Jiangsu Cancer Hospital & Jiangsu Institute of Cancer Research & The Affiliated Cancer Hospital of Nanjing Medical University, No. 42, Baiziting, Nanjing, 210009 China; 2 Key Laboratory of Environmental Medicine Engineering, Ministry of Education, School of Public Health, Southeast University, Nanjing, Jiangsu China

**Keywords:** Docetaxel, Body surface area, Pharmacokinetics, Dosage

## Abstract

**Background:**

Docetaxel (DTX) is a widely used anti-tumour drug, and its dosage is solely determined by body surface area (BSA). Adverse events, such as neutropenia or unsatisfied efficacy, likely occur because of differences in the pharmacokinetics (PK) and pharmacodynamics of patients. Thus, a feasible dosage adjustment method is needed.

**Methods:**

A total of 209 eligible patients who provided consent were enrolled and randomised into two groups to receive the BSA- and PK-guided dosage adjustments of DTX-based chemotherapy (3 weeks per cycle). The AUC of DTX was detected, and the therapeutic window for Chinese patients was determined. The proportion of patients within the therapeutic window was evaluated. Neutropenia was examined in accordance with the toxicity grading standard suggested by the World Health Organisation. Tumour response was assessed in accordance with Response Evaluation Criteria in Solid Tumors version 1.1. The primary endpoint was the incidence of neutropenia, and the secondary endpoints were disease control rate (DCR) and 3-year survival rate.

**Results:**

The therapeutic window for Chinese patients was 1.7–2.5 mg·h/L. The proportion of patients within the therapeutic window was 63.89% versus 28.33% (*P *< 0.0001), and the incidence of neutropenia was 68.33% versus 38.89% (*P *= 0.001) in the experimental group versus the control group in the sixth cycle, respectively. DCR was 72% versus 85% (*P *= 0.018) in the control group versus the experimental group. The 3-year survival rate of the PK group was significantly higher than that of the BSA group (*P *= 0.034).

**Conclusions:**

The PK-guided dosage adjustment of DTX could significantly increase the proportion of patients within the therapeutic window, decrease the incidence of neutropenia and increase the DCR and the 3-year survival rate. The PK-guided dosage adjustment based on the dynamic monitoring of AUC could be a useful method for oncologists to improve individualised treatment options, optimise drug efficacy and reduce drug toxicity.

## Background

Docetaxel (DTX) is a paclitaxel-based antineoplastic drug that plays an antineoplastic role by interfering with the microtubule network necessary for cell mitosis and mitotic interphase [1]. DTX can bind to free tubulin, promote tubulin assembly into stable microtubules and inhibit its depolymerisation; this mechanism of action results in microtubule bundle malfunction and microtubule immobilisation, thereby inhibiting cellular mitosis [2]. DTX is widely used in the treatment of breast cancer, ovarian cancer, non-small cell lung cancer, gastric cancer, pancreatic cancer, melanoma and other cancer types [3, 4].

Similar to the dosage of other chemotherapeutic reagents, DTX dosage is calculated solely on the basis of a patient’s body surface area (BSA). However, clinical data suggest that administering patients with the same dosage of DTX results in significant pharmacokinetic (PK) differences because of internal or external factors, such as genomic composition, physiological status, genetic characteristics and living habits [5]. Remarkable individual pharmacodynamic differences are also observed because of the narrow effective therapeutic range (therapeutic window) of DTX blood concentration [6]. A low drug concentration may elicit unsatisfactory therapeutic effects, whereas a high drug concentration likely leads to increased toxicity and adverse effects, which are the main reasons for the high toxicity of chemotherapeutic drugs and treatment failure [7]. Therefore, a feasible dosage adjustment method for each patient is needed.

Neutropenia is one of the main dose-limiting toxic effects of DTX. It can induce neutropenic fever, reduce dosage and result in early treatment discontinuation [7]. The risk factors of neutropenia include old age, low weight, high DTX dose and numerous chemotherapy cycles [8].

The area under a curve (AUC) of blood concentration is an important index for evaluating the absorption and PK of drugs in vivo. Neutropenia is closely correlated with the AUC of DTX amongst patients receiving DTX-based chemotherapy [9]. Bruno et al. [10] statistically analysed the PK data of 640 patients receiving DTX-based chemotherapy and found that AUC is statistically correlated with the incidence of neutropenia. Therefore, we hypothesised that dosage adjustment based on the dynamic monitoring of the AUC of DTX for each patient might be a useful method for oncologists to improve individualised treatment options, optimise drug efficacy and reduce drug toxicity [11].

In the present study, 209 patients who had solid tumours and received DTX-based chemotherapy were enrolled and randomised into a PK-directed DTX dosage adjustment group (PK group or experimental group) and a BSA-based dosage group (BSA group or control group). An optimised therapeutic window for the Chinese population was explored and defined, and the proportion of patients within this therapeutic window was evaluated. The objective response rate (ORR), disease control rate (DCR), 3-year survival rate and neutropenia incidence were also evaluated.

## Methods

### Ethical statements

The Ethical Committee of Jiangsu Cancer Hospital approved the study, and all the patients signed an informed consent for the use of their personal data for research purposes. This study was conducted in accordance with the Declaration of Helsinki, Guidelines for Good Clinical Practice and the local laws and regulations of China.

### Study design, patient eligibility and treatment

This work was a single-centre, prospective, randomised controlled study.

The eligibility criteria of the study were as follows: (1) male or female aged over 18 years; (2) histologically confirmed solid tumours that could not be subjected to surgery or radiotherapy; (3) Eastern Cooperative Oncology Group score of 0–2, life expectancy of not less than 12 weeks; and (4) organ function level that met the following requirements: absolute neutrophil count ≥ 1.5 × 10^9^/L, platelet count ≥ 75 × 10^9^/L and haemoglobin ≥ 90 g/L; serum total bilirubin of less than 1.5 times the upper limit of the normal value; aspartate aminotransferase and alanine aminotransferase of less than 2.5 times the upper limit of the normal value and serum creatinine of less than 1.5 times the upper limit of normal value; or creatinine clearance rate of more than 50 mL/min. The exclusion criteria were as follows: (1) received surgery or radiotherapy in the past 3 months; (2) abnormal liver, kidney, heart function and blood routine; and (3) patients with insufficient compliance in this experiment or receiving other treatments during the treatment course.

Patients who consented to the study were randomly divided into the experimental group (*n *= 109, also referred to as the PK group) and the control group (*n *= 100, also referred to as BSA group). All eligible patients received DTX-based chemotherapy 3 weeks per cycle. Chemotherapy did not exceed six cycles. In accordance with the patient’s physical condition, dosage was calculated on the basis of a BSA of less than 75 mg/m^2^. The drug was administered via an intravenous drip and completed within 1 h. From the second cycle, the PK group received dosage adjustment based on the PK parameter AUC of DTX. The BSA group continued to receive BSA-guided dosage. If the disease progressed or patients developed intolerable adverse reactions, treatment was discontinued, and patients received the best support of care.

### AUC calculation

After 60 min of intravenous drip, 2–3 mL of blood was collected as samples 1 and 2, respectively, with an accurate recording of collection time. The blood samples were stored in a refrigerator at 2 °C–8 °C within 10 min. Plasma was isolated through centrifugation within 12 h. The blood concentration of DTX was detected using a DTX assay kit (Jiangsu Changxing Medical Technology Co., Ltd.). AUC was calculated on the basis of DTX dosage, start and end time of intravenous drip, blood sample collection time and DTX blood concentration by using the population PK model software provided by Saladax Company.

### Clinical assessments

Efficacy was evaluated every two chemotherapy cycles. Response was assessed in accordance with the Response Evaluation Criteria in Solid Tumors (RECIST 1.1). DTX-related neutrophils were classified in accordance with the toxic grading standard suggested by WHO.

### Outcomes and endpoints

This study was designed to evaluate the effects of AUC-based dosage adjustment on clinical outcomes, such as the population rate within the therapeutic window and the incidence rate of neutropenia. The primary endpoint was the incidence rate of grades 3 and 4 neutropenia. The secondary endpoint was disease control rate (DCR). ORR was defined as the percentage of patients with a complete response (CR) or a partial response (PR). DCR was defined as the percentage of patients with a CR, a PR or a stable disease (SD).

### Statistical methods and sample size calculation

Data were analysed with SPSS 20.0, and measurements were expressed as mean (± standard deviation, SD). P < 0.05 indicated significant differences (*, P < 0.05; **, P < 0.01; ***, P < 0.001). The incidence rate of neutropenia between the two groups was compared via a Chi square test. DCR was compared via a Mantel–Haenszel test, and survival was examined with Kaplan–Meier analysis.

## Results

### Patient characteristics

Patients diagnosed with solid tumours and scheduled to receive DTX-based chemotherapy in our hospital from January 2015 to December 2016 were screened, and 209 patients were enrolled in this study. The patient baseline characteristics are summarised in Table 1. The 209 eligible patients who consented to participate (59 cases of non-small cell lung cancer, 9 cases of nasopharyngeal carcinoma, 42 cases of breast cancer, 34 cases of oesophageal cancer, 42 cases of prostate cancer and 23 cases of gastric cancer) were randomised into PK (*n *= 109) and BSA (*n *= 100) groups. Of the 209 randomised patients, 130 (62%) were male and 79 (38%) were female. The patients were 20–84 years old (57 ± 12). The baseline characteristics were similar between experimental and control groups and had no significant differences. All patients received treatment as allocated.

**Table 1:** Patient characteristics

Variable	Experimental (*n* = 109)	Control (*n* = 100)	*P*
Median age, years (SD)	54.84 (13.44)	59.08 (10.37)	
Age group			
< 65	83 (55)	68 (45)	0.2172
≥ 65	26 (45)	32 (55)	
Sex			
Male	69 (63)	61 (61)	0.7761
Female	40 (37)	39 (39)	
Type of cancer			
Nasopharyngeal carcinoma	7 (78)	2 (22)	0.3101
Lung cancer	26 (44)	33 (56)	
Prostate cancer	21 (50)	21 (50)	
Breast cancer	25 (60)	17 (40)	
Oesophageal cancer	16 (47)	18 (53)	
Gastric cancer	14 (61)	9 (39)	
Line of therapy			
1	62 (57)	52 (52)	0.6755
2	28 (26)	26 (26)	
> 2	19 (17)	22 (22)	
ECOG PS			
0–1	93 (85)	88 (88)	0.5701
2	16 (15)	12 (12)	

Data presented as No. (%) unless otherwise indicated

*ECOG PS* Eastern Cooperative Oncology Group performance status

### AUC of DTX amongst all patients

In the 100 patients in the BSA group, the AUC of DTX was 0.5–5.3 (1.96 ± 0.81) mg h/L in the first cycle, 0.7–4.7 (1.90 ± 0.66) mg h/L in the second cycle, 0.7–3.6 (1.89 ± 0.58) mg h/L in the third cycle, 1.5–3.7 (2.35 ± 0.58) mg h/L in the fourth cycle, 1.3–4.6 (2.68 ± 0.72) mg h/L in the fifth cycle and 1.8–4.2 (2.85 ± 0.59) mg h/L in the sixth cycle. In the 109 patients in the PK group, the AUC of DTX was 0.5–5.1 (1.87 ± 0.87) mg h/L in the first cycle, 0.6–3.7 (1.81 ± 0.62) mg h/L in the second cycle, 1.1–3.6 (2.11 ± 0.5) mg h/L in the third cycle, 1.2–3.1 (2.12 ± 0.42) mg h/L in the fourth cycle, 1.2–3.1 (2.1 ± 0.43) mg h/L in the fifth cycle and 1.4–3.1 (2.28 ± 0.43) mg h/L in the sixth cycle.

**Fig. 1:**
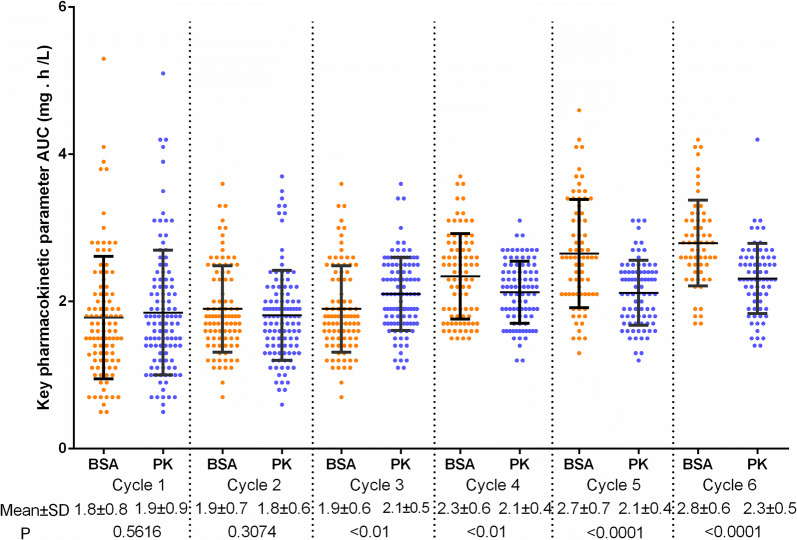
Comparison of the key PK parameters of AUC of each treatment cycle between BSA and PK groups. From the third treatment cycle, the AUC of paclitaxel in the PK group was significantly lower than that in the BSA group

In the BSA group, the mean AUC of DTX constantly increased with the prolonged chemotherapy cycle. This result might be related to decreased tolerance and metabolism rate. The AUC was dispersed in all the cycles. In the PK group, the AUC was 0.5–5.1 (1.87 ± 0.87) mg h/L after BSA-based dosage was administered in the first cycle, which was as dispersed as in the control group, but being less dispersed after the cycles of dosage adjustment as indicated by the decreased standard derivation (Fig. 1 and Table 2). The proportions of patients within the therapeutic window (1.7–2.5 mg h/L) were 36.00% versus 36.70% (*P *= 0.9166), 41.00% versus 50.46% (*P *= 0.1705), 50.00% versus 61.90% (*P *= 0.0928), 48.24% versus 62.64% (*P *= 0.0546), 36.62% versus 71.08% (*P *< 0.0001) and 28.33% versus 63.89% (*P *< 0.0001) in the control group versus the experimental group from cycles one to six, respectively (Table 2).

**Table 2:** Proportion of patients within the therapeutic window

	Experimental (PK) group	Control (BSA) group	P value
Cycle 1	36.70 (0.87)	36.00 (0.81)	0.9166
Cycle 2	50.46 (0.62)	41.00 (0.66)	0.1705
Cycle 3	61.90 (0.50)	50.00 (0.58)	0.0928
Cycle 4	62.64 (0.42)	48.24 (0.58)	0.0546
Cycle 5	71.08 (0.43)	36.62 (0.72)	< 0.0001
Cycle 6	63.89 (0.43)	28.33 (0.59)	< 0.0001

Data presented as  % (SD) unless otherwise indicated; *SD* standard derivation of AUC

### AUC of DTX amongst patients with different cancer types

**Fig. 2:**
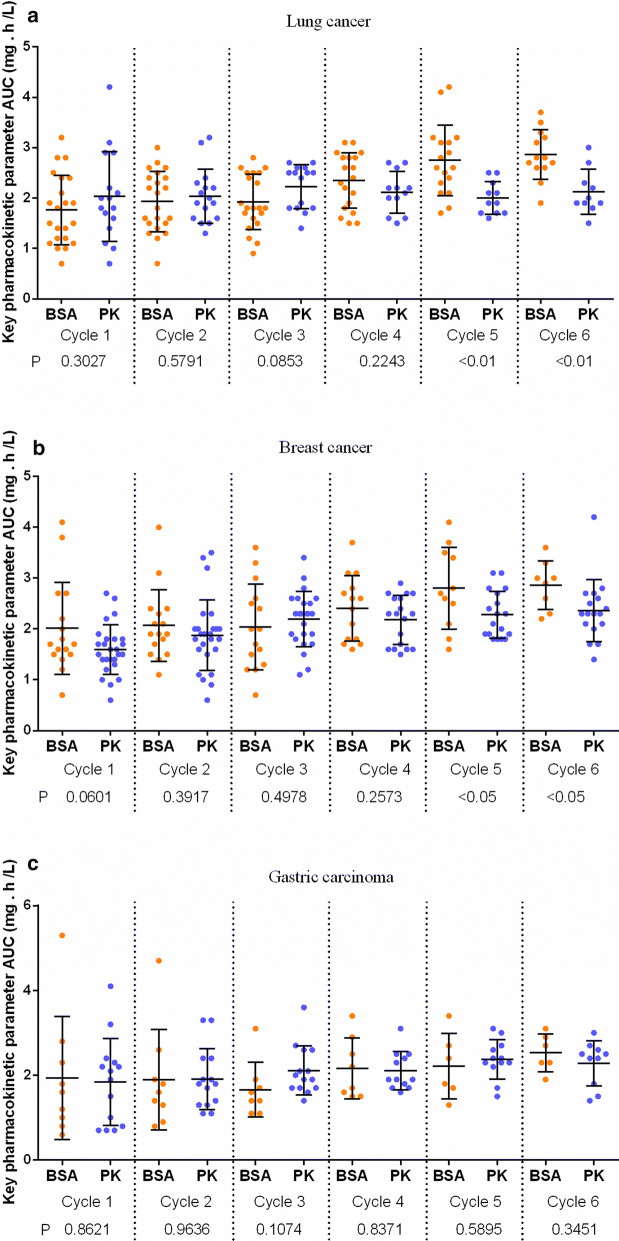
Comparison of the AUC of each treatment between BSA and PK groups in lung, breast and stomach cancers

The AUC of paclitaxel amongst patients with lung cancer, breast cancer and gastric cancer under BSA- and PK-guided medication was further analysed. In lung cancer and breast cancer, the AUC of cycles 5 and 6 of the PK group was significantly lower than that of the BSA group, but the difference in the AUC of cycles 1–4 between the PK and BSA groups was not significant (Fig. 2). In gastric carcinoma, no significant difference in AUC was observed between PK and BSA groups for cycles 1–6 (Fig. 2).

### Incidence of neutropenia

**Fig. 3:**
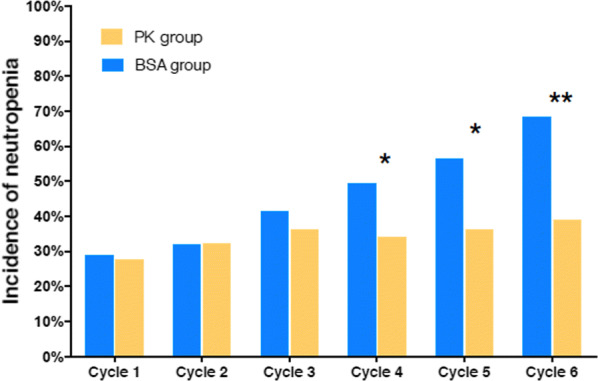
Incidence of neutropenia amongst the PK and BSA groups. From the fourth treatment cycle, the incidence of grade 3 and above neutropenia in the PK group was significantly lower than that in the BSA group. *, *P *< 0.05 compared with the BSA group; **, *P *< 0.01 compared with the BSA group

The incidence rates of neutropenia with grade 3 and above were 29.00% versus 27.52% (*P *= 0.814), 32.00% versus 32.11% (*P *= 0.986) and 41.30% versus 36.19% (*P *= 0.467) in the control group versus the experimental group in the first, second and third cycles of chemotherapy, respectively. No significant differences were observed. In the fourth, fifth and sixth chemotherapy cycles, the incidence rates were 49.41% versus 34.07% (*P *= 0.039), 56.34% versus 36.14% (*P *= 0.012) and 68.33% versus 38.89% (P = 0.001) in the control group versus the experimental group (Fig. 3 and Table 3). From the fourth cycle, the incidence rate of neutropenia between the control and experimental groups began to show significant differences. The toxicity of the PK group with dosage adjustment was lower than that of the BSA group during the whole chemotherapy cycle. This result indicated that the PK-guided dosage adjustment of DTX could effectively control the toxicity of chemotherapy and reduce its side effects.

**Table 3:** Incidence rate of neutropenia with grade 3 and above

	Experimental (PK) group	Control (BSA) group	*P*
Cycle 1	27.52 (30/109)	29.00 (29/100)	0.814
Cycle 2	32.11 (35/109)	32.00 (32/100)	0.986
Cycle 3	36.19 (38/105)	41.30 (38/92)	0.467
Cycle 4	34.07 (31/91)	49.41 (42/85)	0.039
Cycle 5	36.14 (30/83)	56.34 (40/71)	0.012
Cycle 6	38.89 (28/72)	68.33 (41/60)	0.001

Data presented as  % (No. of grade 3 and above AE/Total patients) unless otherwise indicated. *AE* adverse effects

### Efficacy

**Fig. 4:**
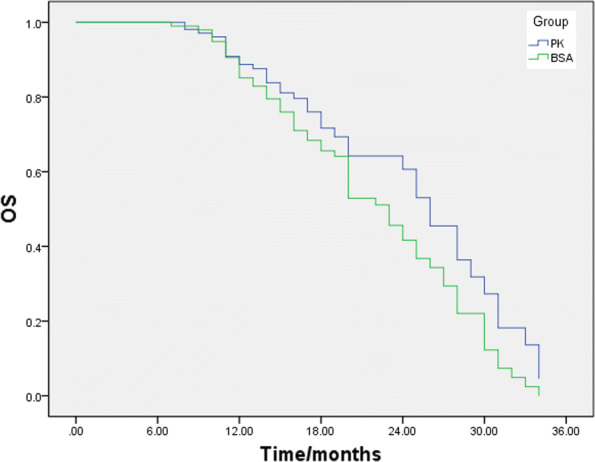
Three-year survival rate of patients receiving BSA-guided dosage adjustment versus PK-guided dosage adjustment. The difference between the two groups was significant (67.8% in the PK group vs. 39.0% in the BSA group, *P *= 0.034)

Efficacy is listed in Table 4. After data collection was completed in March 2018, 34 of 100 (34%) patients in the control group and 40 of 109 patients (37%) in the experimental group reached the best overall response of PR, 38 of 100 (38%) patients in the control group and 53 of 109 patients (49%) in the experimental group achieved the best overall SD response, and 28 of 100 (28%) patients in the control group and 16 of 109 patients (15%) in the experimental group achieved the best overall PD response. ORR and DCR were 34% versus 37% (*P *= 0.68) and 72% versus 85% (*P *= 0.018) in the control group versus the experimental group, respectively. The efficacy of the PK group was significantly higher than that of the control group. The 3-year survival rate of the PK group was significantly higher than that of the BSA group (67.8% in the PK group vs. 39.0% in the BSA group, *P *= 0.034), indicating that the PK-guided dosage adjustment of DTX significantly affected the improvement of the survival rate (Fig. 4).

**Table 4:** Efficacy evaluation

	PK group (*n *= 109)	BSA group (*n *= 100)
CR	0 (0.00)	0 (0.00)
PR	40 (36.70)	34 (34.00)
SD	53 (48.26)	38 (38.00)
PD	16 (14.68)	28 (28.00)

Data presented as No. (%) unless otherwise indicated. *CR* complete recession, *PR* partial recession, *SD* stable disease, *PD* progressed disease

## Discussion

This single-centre, prospective and randomised controlled trial amongst patients who had solid tumours and received DTX-based chemotherapy met its primary endpoint of the population rate within the therapeutic window, the incidence rate of neutropenia and the secondary endpoint of DCR. This study also explored the optimised AUC range for Chinese patients.

Many studies have shown that the data regarding the statistical correlation between BSA and AUC of DTX are insufficient; other factors also affected the clearance for doxorubicin, such as concomitant drugs, infusion duration and sex. In addition, the above studies mainly focused on the solid tumors, not the other types of cancer [12, 13]. Similar to most chemotherapeutic agents, DTX is administered in accordance with BSA. However, two patients with the same BSA are often encountered clinically, and their efficacy and adverse reactions may be completely different when they receive the same dose of the same chemotherapeutic drug. This difference is probably attributed to the varying PK amongst individuals. Studies have shown that neutropenia is associated with an excessive BSA, a low BMI and a long chemotherapy cycle [14]. Therefore, patients who receive BSA-based dosage may have severe toxic reactions because of high dosage, leading to adverse events. In addition, patients rarely have an opportunity to use high doses to achieve their maximum tolerated dose in a chemotherapy course. Thus, a considerable proportion of patients do not reach the most effective dose of DTX. Remarkable PK differences exist between individuals whose doses are dependent on BSA, and achieving an optimal AUC is difficult [15]. The relationship between the bioavailability of drugs and AUC is observed in patients after the BSA-based treatment was administered, and the difference in the plasma concentration of DTX between individuals is seven times [15]. These differences reflect the variations in the drug clearance rate between individuals and indicate the need for an appropriate method of determining doses.

With the application of reversed phase high-performance liquid chromatography, high-performance liquid chromatography–tandem mass spectrometry and novel nano-enhanced immunoturbidimetric assay in clinical practice, physicians can use PK as a clinical medication guide. The amount of medicine absorbed in blood circulation after a single dose can be expressed using AUC, which reflects the relative amount of medicine entering the blood circulation. The range of the DTX therapeutic window is not unified because of the differences in AUC detection and calculation methods and the variety of toxicity criteria amongst research groups. Minami [16], Ozawa [17] and Engels [12] showed that the optimal AUC range of DTX for European and American patients is 2.5–3.7 mg h/L. However, the optimal AUC range for Asians, especially for the Chinese population, is not well defined. In the present study, the average AUC of DTX was 1.8 mg h/L, and the AUC of the majority of patients was 1.7–2.5 mg h/L. Therefore, the therapeutic window in this study was set to 1.7–2.5 mg h/L. This difference could be related to genetic background, living habits and other factors affecting the PK of DTX. With limited data and sample size, the mechanism could not be explained yet, and this range was preliminary. More data should be collected to validate and modify the feasible target range for Asian patients.

In this study, the patients from the experimental group received PK-guided dosage adjustment. From the second to the sixth cycles, the dosage of the PK group was adjusted in accordance with the AUC of the previous cycle. In the sixth cycle, the AUC in the PK group was 1.4–3.1 (2.28 ± 0.43) mg h/L and less dispersed compared with that of the BSA group with a range of 1.8–4.2 (2.85 ± 0.59). The proportion of the patients in the therapeutic window increased after each cycle of adjustment. After six cycles of adjustment, their proportion reached 63.89% versus 28.33% (P < 0.0001) in the experimental group versus the control group. Thus, PK-guided dosage adjustment can effectively optimise DTX dosage. The incidence of neutropenia (grade 3 or above) in the experimental group was also significantly lower than that of the control group after three cycles of dosage adjustment. PK-guided dosage adjustment significantly increased the DCR and improved the survival rate of patients. Therefore, PK-guided dosage adjustment based on AUC detection could be a potential therapeutic option to optimise efficacy, reduce toxicity and improve the living quality of patients.

The effects of PK-guided medication on patients with lung cancer, breast cancer and gastric cancer were investigated. At the late stage of chemotherapy for lung cancer and breast cancer, the AUC of PK-guided therapy was significantly lower than that of BSA-guided one. In the whole course of chemotherapy for gastric cancer, the difference in AUC between the PK- and BSA-guided drugs was not significant. Therefore, the PK guidance for DTX was in applicable to all malignant tumours. The value of PK-guided DTX for patients with lung cancer and breast cancer was higher than that for patients with gastric cancer.

This study has several limitations. The proportion of the patients within the therapeutic window and the incidence of neutropenia significantly differed after several cycles of adjustment. However, the first two cycles were not significant, indicating that dosage adjustment should be further optimised. The PK-guided dosage adjustment based on AUC detection did not prolong the patients’ life in our study although their quality of life significantly improved. With limited data, the difference in the therapeutic window between Chinese, European and American populations could not be explained. Further studies are needed to answer these questions.

## Conclusion

The PK-guided dosage adjustment of DTX could significantly increase the proportion of patients within the therapeutic window, decrease the incidence of neutropenia and increase the DCR and the 3-year survival rate. The PK-guided dosage adjustment based on the dynamic monitoring of AUC could be a useful method for oncologists to improve individualised treatment options, optimise drug efficacy and reduce drug toxicity.

## Data Availability

The datasets used and analyzed during the current study are available from the corresponding author on reasonable request.

## Abbreviations

AUC: Area under a curve
BSA: Body surface area
CR: Complete response
DCR: Disease control rate
DTX: Docetaxel
ORR: Objective response rate
PK: Pharmacokinetics
PR: Partial response
SD: Stable disease
